# Plaster Burn: Challenge to Plastic Surgeon

**Published:** 2018-05

**Authors:** Varun Singh Chauhan, Mohd Altaf Mir

**Affiliations:** 1 Department of Plastic and Reconstructive Surgery, PGIMER and Dr. RML Hospital, India;; 2 Department of Plastic and Reconstructive Surgery, AMU, India

**Keywords:** Plaster, Burn, Skin, Grafting, Flap

## Abstract

**BACKGROUND:**

The thermal burn is accidental and also is the hidden and worst complication of medical plaster application. This study evaluated clinical and aetiological profile and severity of plaster burns.

**METHODS:**

In Department of Burns, Plastic and Reconstructive Surgery from 1^st^ August 2014 to 31^st^ December 2015, six patients with plaster burn were assessed for total body surface area and depth of burn. The wounds were cultured and dressed with moist dressings daily till the surgical procedure and satisfactory healing.

**RESULTS:**

The youngest patient was 10 and oldest 65 years (mean age: 40.20±6.67 years, 4 males and two females with ratio of 2:1). Three patients sustained plaster burn injury accidentally at home and 3 developed burn after medical application of plaster. The hands burns were observed commonly in accidental plaster burns, while ankle was often involved in iatrogenic plaster burns. The iatrogenic burns were mostly deep in thickness varying 2^nd^ to 4^th^ degree, while accidental burns were often 2^nd^ degree. Superficial 2^nd^ degree burns were managed conservatively, and deep 2^nd^ degree burns were skin grafted. Fourth degree burn was managed with reverse sural flap alone and another with vacuum-assisted closure followed by reverse sural flap and skin grafting.

**CONCLUSION:**

Plaster burn is still a challenge to plastic surgeon and it is advised for applying casts to utilize all available means to limit the amount of casting material provided. It can be accomplished without compromising the cast strength to minimize the risk of thermal injury when applying plaster or composite casts.

## INTRODUCTION

Burn is still considered devastating in emergency medicine leading to physical and psychological disabilities and has an increasing mortality and morbidity.^1^ Mainstay of post-burn reconstruction is release and split skin grafting. Pedicle flaps were shown to be cumbersome, to need several procedures and hospitalization and free flaps are still technically demanding and facilities are not available everywhere.^2^ Plaster of Paris is a useful material with many properties. It is used in art, architecture, fireproofing, and medical applications. The name POP was derived from an accident to a house which was built from a deposit of gypsum, near Paris. The house was burnt down and when rain fell on baked mud of the floors it was noted that the footprints in the mud set rock hard. Burns from plaster of Paris is an uncommon complication due to improper use of this material.^3^

Plaster of Paris is classified as a hazardous substance. When mixed with water, this material hardens and then slowly becomes hot and raises temperatures as high up to 60 degrees centigrade.^4^^-^^7^ Superficial to deep thickness body surface area burns can occur at much lower temperatures, perhaps as low as 45 degrees centigrade, if contact is prolonged.^4^^-^^6^ It is a challenge to plastic surgeon because patient presents late to plastic surgeon. It is seen commonly in children and older age group.

## MATERIALS AND METHODS

This study was conducted in the Postdoctoral Department of Burns, Plastic and Reconstructive Surgery of our institution from 1^st^ Aug 2014 to 31^st^ Dec 2015. Six patients with the plaster burn referred to the burns, reconstructive and plastic surgery unit over a period of 17 months. The patients wound were assessed for the percentage of total body surface area and depth of burn. The wound culture sensitivity was done in each patient and wounds were dressed with moist dressings daily till the surgical procedure and or satisfactory healing.

## RESULTS

The youngest patient was 10 years and oldest 65 years with the mean age of 40.20±6.67 years. There were 4 males and 2 females with ratio of 2:1. Three patients sustained plaster burn injury accidentally at home and 3 patients developed burn after medical application of plaster. The hand burns were observed commonly in accidental plaster burns while as ankle was often involved in iatrogenic plaster burns. The iatrogenic burns were mostly deep in thickness varying 2^nd^ degree deep to 4^th^ degree while as in accidental burns were most often 2^nd^ degree burns. Superficial 2^nd^ degree burns were managed conservatively, and deep 2^nd^ degree burns were skin grafted. In only one case (case 6) we observed partial loss of graft. One 4^th^ degree burn heal was managed with reverse sural flap alone and another with vacuum-assisted closure followed by the reverse sural flap and skin grafting. The patients’ clinical profile is summarized in Table 1.

**Table 1:** Clinical profile of plaster burns.

**Case **	**Age/sex**	**Site **	**Reason for plaster application**	**Depth of plaster burn**	**Management **	**Complication**
1	65/F	Right heel and ankle	Fracture of femur, both bone fracture upper part right leg	4^th^ degree (Figure 1a and b)	Reverse sural flap	Nil
2	11/M	Right foot dorsal aspect	RTA with closed fracture of metatarsal of right foot	2^nd ^degree deep	STSG	Nil
3	10 /M	Left hand burn	Accidental	2^nd^ degree superficial	Conservative	Nil
4	20/F	Right hand	Accidental	2^nd^ degree superficial (Figure 3)	Conservative	Nil
5	45/M	Left heel	Fracture of upper end of tibia left leg	4^th^ degree (Figure 2a, b and c)	VAC followed by reverse sural Flap and and STSG	Nil
6	55/M	Right foot	Accidental	2^nd^ Degree deep (Figure 4a and b)	STSG	Partial graft loss

**Fig. 1:**
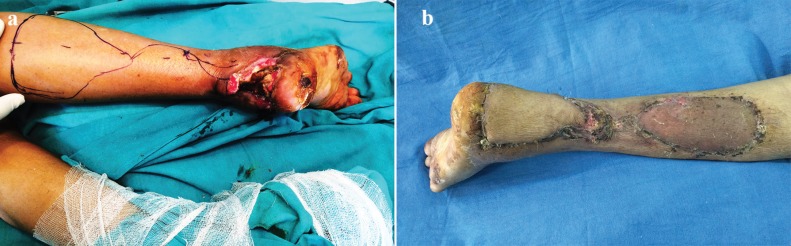
a: Preoperative photograph shows 4th degree plaster burn right ankle in case 1. b: Postoperative photograph after reconstruction with reverse sural sural flap in case 1.

**Fig. 2:**
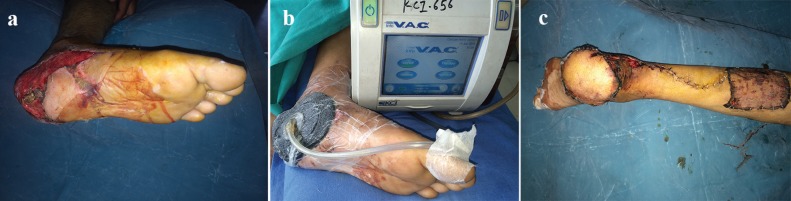
a: Preoperative photograph shows 4th degree plaster burn left heal in case 5. b: Preoperative photograph of 4th degree plaster burn left heal with VAC in case 5. c: Postoperative photograph after reconstruction with reverse sural sural flap in case 5.

**Fig. 3:**
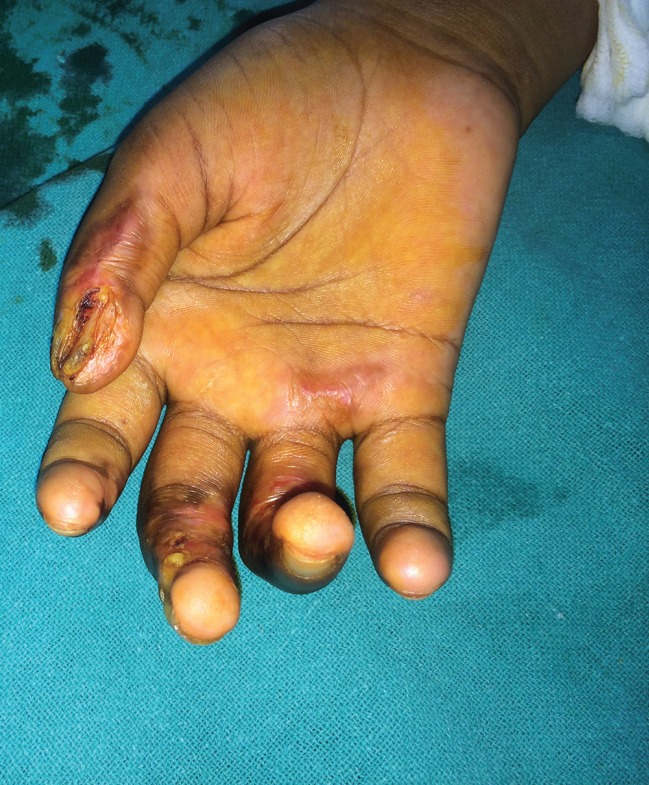
Photograph shows healing second degree superficial burn of right hand in case 4.

**Fig. 4:**
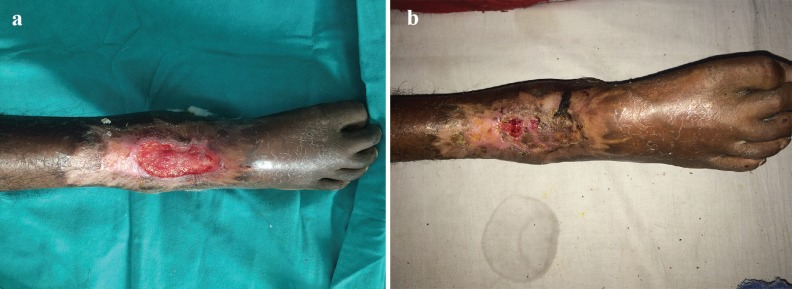
a: Preoperative photograph shows 2nd degree plaster burn right foot dorsum in case 6. b: Post STSG photograph of right foot dorsum shows partial loss of skin graft in case 6.

## DISCUSSION

Plaster of Paris is a very useful material. It is used in art, architecture, fireproofing, and medical applications. Plaster of Paris is made by heating gypsum, a process which involves exposing the gypsum to very high temperatures to create calcium sulfate and then grinding it into a fine white powder. When water is added to the powder it quickly rehydrates and the slurry can be molded in a variety of ways. As it sets, a firm matrix is created, which has a smooth solid shape. It is not dangerous, if it is worked with responsibly and should be used with great respect but not fear. Improper use of this may lead to injury to the unaffected part of the body. Improper use of plaster of Paris may lead to burn of the normal tissues of the body.^3^ Burn may extend from superficial to deep burns. Factors that can cause thermal injury are temperature of dip water, cast thickness, use of insulating pillows/blankets while the cast is drying.^4^

Elderly people and young children tend to be more susceptible to burn injury because of decreased skin thickness, decreased reaction time, or decreased cutaneous sensation.^4^ Enclosing any part of the body for making casts using this material is potentially very dangerous, if the thickness of the cast exceeds a few millimetres. Once mixed, the plaster will set rapidly into a solid rigid mass. Anything that is embedded in the plaster may therefore quickly become trapped and exposed to an extreme temperature. carelessness can cause severe burns that may require surgical removal of affected tissue or amputation of digits or a limb. As temperature of the thermal insult increases, the time required to produce a specific injury is decreases substantially.^4^

It was previously demonstrated that plaster could, under the right set of conditions, create temperatures at or above the burn threshold which may lead to thermal injury to normal tissues.^6^^,^^7^ Temperature of dip water should feel slightly cool to the touch. It has been emphasized that when the cast rolls are soaked in hot water, they absorb water at a slower rate than when they are soaked in cold water.^3^ Also, if the cast rolls are dipped in water for a short period of time, they absorb less water. When less water is present in the cast rolls, the temperature of the cast increases. The specific heat capacity of water of 4,190 J/kg K is very high, which means the more water is present, the more of a cooling effect the water has on the temperature of the cast.^8^ It was shown that pressure applied during the molding of casts results in a significant increase in temperature at the side where the mold was applied.^9^ It is important to limit the amount of casting material, provided it can be accomplished without compromising cast strength.^10^

Plaster burn is a challenge to plastic surgeon as most of the patients were initially managed by the concerned doctor later they are referred to plastic surgeon when the injury became horrible .It is advised for applying casts to utilize all available means to minimize the risk of thermal injury when applying plaster or composite casts to children or adults: room temperature, clean, dip water; minimum required thickness of plaster; avoid covering the cast with blankets while it is drying; avoid setting the freshly applied cast on an insulating pillow. Plaster burn is a challenge to plastic surgeon and it is advised for applying casts to utilize all available means to limit the amount of casting material provided it can be accomplished without compromising the cast strength to minimize the risk of thermal injury when applying plaster or composite casts.

## CONFLICT OF INTEREST

The authors declare no conflict of interest.

## References

[1] Mohammadi AA, Tohidinik HR, Zardosht M, Seyed Jafari SM (2016). Self-burns in Fars Province, Southern Iran. World J Plast Surg.

[2] Panse N, Sahasrabudhe P, Bhatt Y (2012). Use of local perforator flaps for post burn reconstruction. Word J Plast Surg.

[3] Haasch K (1964). Burns under the plaster cast. Hefte Unfallheilkd.

[4] Rolf D, Burghardt, John G (J Child Orthop). Anderson. Exothermic properties of plaster–synthetic composite casts.

[5] Feller I, James MC (1982). Burn epidemiology: focus on youngsters and the aged. J Burn Care Rehabil.

[6] Gannaway JK, Hunter JR (1983). Thermal effects of casting materials. Clin Orthop Relat Res.

[7] Lavalette R, Pope MH (1982). Setting temperatures of plaster casts. J Bone Joint Surg Am.

[8] Callahan DJ, Carney DJ, Walter ND (1986). The effect of hydration water temperatures on orthopaedic plaster cast strength. Orthopedics.

[9] Deignan BJ, Iaquinto JM, Eskildsen SM, Woodcock CA, Owen JR, Wayne JS, Kuester VG (2011). Effect of pressure applied during casting on temperatures beneath casts. J Pediatr Orthop.

[10] Callahan DJ, Daddario N, Williams S, Walter NE (1986). Three experimental designs testing orthopaedic casting material strength. Orthopedics.

